# The modified Koyanagi hypospadias repair for the one-stage repair of proximal hypospadias

**DOI:** 10.4103/0970-1591.40617

**Published:** 2008

**Authors:** Venkata R. Jayanthi

**Affiliations:** Section of Urology, Columbus Children's Hospital, Columbus, OH 43205, USA

**Keywords:** Chordee, hypospadias, intersex

## Abstract

Perineal and penoscrotal hypospadias were often managed by two-stage urethroplasty with variable results and significant number of these may need third surgery. Though modified Koyanagi one-stage repair has a learning curve, it has all the advantages of two-stage repair. The aim was to review the results of modified Koyonagi repair from the literature and our own centre experience.

## INTRODUCTION

To state the obvious, proximal hypospadias is one of the most challenging conditions to correct. The multiple numbers of procedures that have been described over the years is indicative of the fact that no procedure has been universally acceptable or successful. Many have chosen to perform staged procedures since this has the advantage that the varied anatomical issues can be fixed sequentially with different aspects of the problem being tackled in time. A disadvantage of this approach is that by necessity patients undergo at least two and often more procedures.

The standard two-stage approach involves initial correction of penile curvature along with preparation of a ventral bed of tissue, whether this is composed of transposed flaps of prepuce or grafts of preputial skin, or buccal mucosa. This neourethal plate can then be tubularized at a second setting. The two-stage approach may be the most common method of correcting proximal hypospadias, in part because it is reliable and relatively easy. However, this approach inherently requires every child to undergo two procedures with many requiring a third or more for complications that may develop.

Koyanagi described a procedure whereby lateral flaps of penile shaft skin, in continuity with preputial skin can be brought ventrally and tubularized allowing a one-stage correction of severe hypospadias.[1] The procedure as initially described did have a relatively high-complication rate, in part because no major attempt was made to preserve the blood supply of the skin flaps. Koff *et al*. described a modification of the technique in which the vascularity of the flaps was maintained resulting in a reduced complication rate.[2]

In essence, the Koyanagi technique can simply be described as a two-stage hypospadias repair completed in one-stage. The first step in the procedure involves correction of penile curvature. The second step involves mobilizing lateral skin flaps from the tissue that would have been brought ventrally during a planned first-stage procedure. These skin flaps are composed of the penile skin that would have been tubularized during the second stage.

A major advantage of the technique is that all major dissection is performed in virgin, untouched tissue allowing neourethral reconstruction without any scar tissue affecting vascularity. It has the added advantage that the dissection needed permits simultaneous repair of associated penoscrotal transposition, if present. Thus, all aspects of severe proximal hypospadias can be corrected at one setting.

## SURGICAL TECHNIQUES

### Patient selection

Patients who have proximal hypospadias associated with such significant penile curvature that urethral plate division will be required may be appropriate candidates. Patients with minimal curvature and a healthy urethral plate (essentially those with penoscrotal hypospadias and minimal chordee) may be better served using techniques allowing urethral plate preservation. Our preference in these situations is the onlay island flap repair with dorsal plications as needed. Patients with hair follicles immediately adjacent to the meatus may not be good candidates as this parameatal skin typically needs to be incorporated into the repair.

### Preoperative preparation

Nearly all patients undergo preoperative hormonal stimulation. Our protocol involves the use of HCG 250 international units intramuscularly twice weekly for 5 weeks. Alternatively, testosterone may be used if the child has dysplastic or absent testes. Though there is no objective study comparing HCG with testosterone injections, our preference has been HCG because, theoretically, there may be a more prolonged and sustained testosterone secretion at “physiological” levels produced by the child's testes. Preoperative hormonal therapy increases the size of the penile shaft and perhaps most importantly the glans. The penile shaft skin becomes thicker and more vascular facilitating mobilization of the skin flaps. The degree of chordee also subjectively seems to decrease.[3]

### Chordee correction

If one is considering using the Koyanagi technique, one needs to determine from the outset that this is the procedure that will be done. Often surgeons will initially preserve the urethral plate, deglove the penis, and attempt to correct penile curvature with plications and/or urethral plate mobilization. Then if all these measures are inadequate, urethral plate division is carried out. In contrast, if one is considering a Koyanagi procedure, the urethral plate must be divided distally at the very beginning. This allows the vascularity of the lateral parameatal-based flaps, which in part is derived from the proximal urethral plate, to be preserved. This factor again is an indication that the Koyanagi repair is designed for the most severe cases, where it is “obvious” that the urethral plate must be divided.

**Figure 1:**
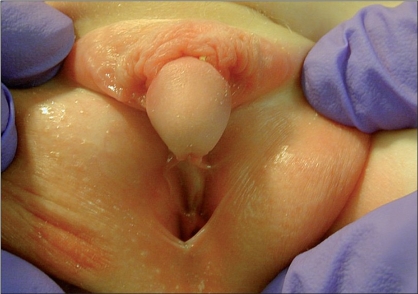
Typical patient amenable to a Koyanagi repair. Severe proximal hypospadias due to intersex condition. Urethral plate must be divided to allow for correction of penile curvature

A circumferential incision is made around the distal shaft, dividing the urethral plate [Figure 1]. This first step is no different than what might be done in the first stage of a planned two-stage repair. The penile skin, in continuity with the urethral plate is mobilized proximally down to the base of the penis. This dissection can be very tedious and it is vital that proximal urethral plate mobilization be carried out into the perineum to ensure that any tethering due to the plate is corrected. There often is atretic, fibrotic spongy tissue fixing the urethral plate to the corporal bodies, and dissection can lead to some bleeding which may be controlled with fine suture ligatures. Once the proximal dissection has been completed, penile curvature may be assessed with an erection test. If significant curvature persists, dorsal plications may be performed. In our experience, preoperative hormonal stimulation often leads to such increased penile growth, that curvature which may persist is minimal and that ventral lengthening procedures are unnecessary.[3]

### Mobilization of the flaps

**Figure 2:**
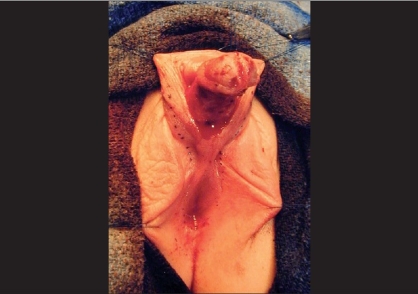
12 month old with mixed gonadal dysgenesis. Perineal opening represents confluence of vagina and urethrea. A distal circumferential incision has been made and the urethral plate dissected proximally for chordee correction. Lateral skin flaps have also been marked out

**Figure 3:**
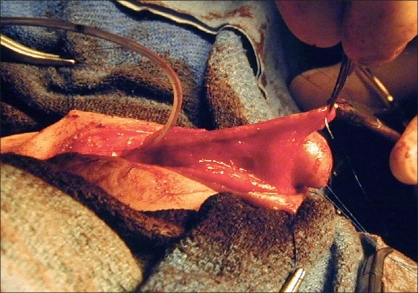
Skin flaps have been mobilized preserving lateral and preputial vascularity

The most important and difficult part of the procedure is the mobilization of the skin flaps. A Y-shaped incision is marked out proximal to the meatus. The “leg” of the Y is in the midline and extends into the scrotum/perineum. The arms of the Y should be directed to each side of the meatus such that extension of the arms will lead to skin flaps composed of lateral penile shaft skin of roughly 7 mm in width [Figure 1]. The lateral skin flaps are marked distally and converge onto the preputial skin. The two lateral flaps essentially meet on the preputial skin, that skin which ordinarily could be mobilized as a transverse preputial island flap. These flaps are then mobilized taking great care to preserve the lateral vascularity [Figures 2 and 3].

At this point, one has essentially dissected one large circumferential flap which may be described as being composed of lateral penile shaft skin as well as prepuce. A buttonhole can be made in the vascular pedicle of the preputial skin. The flap can then be brought ventrally, i.e., the glans is dropped dorsally through the buttonhole. The inner edges of the flaps are sewn together with fine absorbable suture (7-0 polydioxanone). Once this has been accomplished, one has essentially created a neourethral plate.

**Figure 4:**
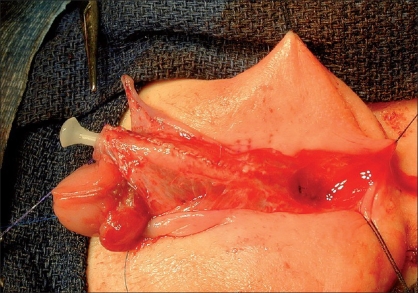
Completed neourethra. The lateral pedicles have been maintained allowing a well vascularised reconstructed urethra

**Figure 5:**
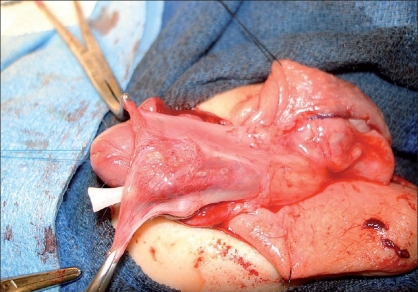
Tunica vaginalis flap has been mobilized from left hemiscrotum and completely covers neourethrav

The outer edges of the skin flaps can then be sutured together in two layers to create the neourethra [Figure 4]. As the skin flaps are planned to be roughly 7 mm in width, the resultant urethra will have a 10-12 mm diameter, approximately 10-12 Fr. This tubularization may be performed over an indwelling 8 Fr catheter. Once the urethra has been constructed, tunica vaginalis may be harvested to lay over the urethra as another layer of coverage [Figure 5].

As in tubularized island flap repairs the neomeatal location can be selected by splitting the glans and forming classic glans wings or by creating a tunnel to the tip, depending on glanular anatomy. Our personal preference is to create a tunnel if adequate ventral glanular tissue is present. Tunneling may lead to better cosmesis with less chance for glanular dehiscence. Once the meatus is constructed, the mucosal collar is wrapped ventrally.

**Figure 6:**
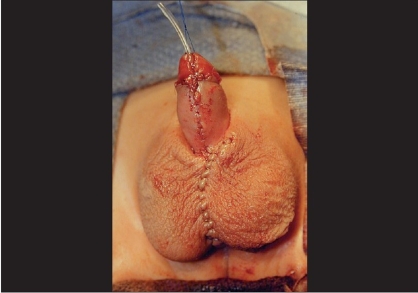
Completed repair. Scrotoplasty corrects bifid scrotum and permits simultaneous repair of penoscrotal transposition

After urethral construction is completed, attention can be shifted to the scrotoplasty and correction of penoscrotal transposition if present. Scrotal flaps may be rotated medially to correct transposition. Subsequent multilayer closure of the scrotum in the midline not only corrects the bifid scrotum, but also provides several layers of coverage over the scrotal/perineal urethra minimizing the risk of proximal fistula formation [Figure 6].

Byer's flaps can then be used to complete skin coverage. A light compression dressing is left in place for several days. The urethral catheter is allowed to drain into a diaper for 14 days. Suprapubic drainage is not necessary. At our center, children are typically discharged home the day of the procedure.

## DISCUSSION

The modified Koyanagi repair is a useful procedure for the one-stage reconstruction of severe hypospadias. It is particularly suited for the most severe forms of hypospadias such as in situations involving intersex conditions such as mixed gonadal dysgenesis or true hermaphroditism.

Complication rates or rather reoperative rates vary from 20 to 50%.[1–6] This seemingly high number must be put into perspective in that if a staged approach is used, 100% of patients will undergo at least two operations with a substantial requiring a third for complications which may develop after urethral tubularization. With the Koyanagi procedure, the majority of patients only require one operation although a sizable number may require a second procedure. Thus using this approach, the total number of operations a cohort of patients with proximal hypospadias may require will be much less than if a planned two-stage approach is used.

Complications typically occur in the penile shaft. Simultaneous scrotoplasty allows for such secure proximal repair that breakdown there is very uncommon. The theoretic problem with overlapping suture lines in the midline can be minimized the placement of a vascularized layer over the repair. Furthermore, fistulae, when they occur usually are relatively small, especially in light of the long length of urethral reconstruction in the first place. Urethral diverticulae can develop if there is an element of meatal stenosis since the penile urethra consists of preputial skin. Importantly, urethral strictures, perhaps the most feared complication are quite rare since no circumferential suture line exists, outside of the meatus and the flaps used for urethral construction are highly vascularized.

## CONCLUSIONS

The modified Koyanagi repair essentially is a two-stage hypospadias repair completed in one operation. Preoperative hormonal stimulation is a vital prerequisite. Despite the high-reoperative rate, it remains our procedure of choice for severe hypospadias. It leads to a nice cosmetic result while simultaneously reducing the overall number of operations most children will require.
